# P2Y1 Receptor Agonist Attenuates Cardiac Fibroblasts Activation Triggered by TGF-β1

**DOI:** 10.3389/fphar.2021.627773

**Published:** 2021-02-17

**Authors:** Geer Tian, Junteng Zhou, Yue Quan, Qihang Kong, Wenchao Wu, Xiaojing Liu

**Affiliations:** ^1^ Laboratory of Cardiovascular Diseases, Regenerative Medicine Research Center, West China Hospital, Sichuan University, Chengdu, China; ^2^ Department of Cardiology, West China Hospital, Sichuan University, Chengdu, China

**Keywords:** P2Y1 receptor, purinergic receptors, cardiac fibroblast activation, cardiac fibrosis, transverse aortic constriction

## Abstract

Cardiac fibroblasts (CFs) activation is a hallmark feature of cardiac fibrosis caused by cardiac remodeling. The purinergic signaling molecules have been proven to participate in the activation of CFs. In this study, we explored the expression pattern of P2Y receptor family in the cardiac fibrosis mice model induced by the transverse aortic constriction (TAC) operation and in the activation of CFs triggered by transforming growth factor β1 (TGF-β1) stimulation. We then investigated the role of P2Y1receptor (P2Y1R) in activated CFs. The results showed that among P2Y family members, only P2Y1R was downregulated in the heart tissues of TAC mice. Consistent with our *in vivo* results, the level of P2Y1R was decreased in the activated CFs, when CFs were treated with TGF-β1. Silencing P2Y1R expression with siP2Y1R accelerated the effects of TGF-β1 on CFs activation. Moreover, the P2Y1R selective antagonist BPTU increased the levels of mRNA and protein of profibrogenic markers, such as connective tissue growth factor (CTGF), periostin (POSTN). periostin (POSTN), and α-smooth muscle actin(α-SMA). Further, MRS2365, the agonist of P2Y1R, ameliorated the activation of CFs and activated the p38 MAPK and ERK signaling pathways. In conclusion , our findings revealed that upregulating of P2Y1R may attenuate the abnormal activation of CFs via the p38 MAPK and ERK signaling pathway.

## Introduction

Cardiac fibrosis caused by complex cellular reprograming process is an independent risk factor of cardiac mortality (Dubey et al., 1997). The process of cardiac fibrosis is described as cardiac morphology disruption, extracellular matrix (ECM) deposition, and cardiac function impaired (Lyon et al., 2015). The main cause of cardiac fibrosis is the activation of cardiac fibroblasts (CFs). TGF-β1 is an important cytokine to mediate CFs proliferation and apoptosis, which is involved in the process of cardiac fibrosis and widely used to induce fibroblasts activation (Sledzińska et al., 2013). However, the underneath molecular mechanism of cardiac fibrosis and CFs activation have not been completely elucidated.

The purinergic receptor family is a type of membrane protein targeted by nucleotides to transmit intracellular signals, and the system is divided into adenosine receptors (P1) and ATP/ADP receptors (P2) (Burnstock, 2018). Till now, four P1 G-protein-coupled receptor subtypes, seven P2X ion channel receptor subtypes, and eight P2Y G-protein-coupled receptor subtypes are recognized (Burnstock, 2017). Evidence reported that four subtypes of P1 receptors are expressed on cardiac fibroblasts and cardiomyocytes, and mediate cardioprotection and modulate the collagen and protein synthesis (Novitskaya et al., 2016). Several P2 receptors (Y1, Y2, Y4, Y6, and Y11) are also expressed on cardiomyocytes and involved in the intercellular synchronization of intracellular Ca^2+^ oscillations in cardiomyocytes (Kim and Woo, 2015). Our previous study revealed that the expression pattern of P2X subtypes, and among them, P2X7R was considered as a critical receptor to promote cardiac fibrosis (Zhou et al., 2020). Among the P2Y family, P2Y1R is a purine-specific receptor that was first isolated from a chick brain and showed a close relationship between P2Y1R and neurological diseases (Carvalho et al., 2019). The recent study reported that P2Y receptors play a crucial role as therapeutic target in myocardial protection during ischemia/reperfusion (Djerada et al., 2017). However, changes in P2Y subtypes expression profile and its function in the process of cardiac fibrosis are still not known.

Therefore, in this study, we explored the expression pattern of P2Y subtypes and postulated that P2Y1R may be a therapeutic target for cardiac fibroblasts activation and cardiac fibrosis.

## Materials and Methods

### Animal Experiments and Transverse Aortic Constriction (TAC) Operation

Male C57BL/6 mice (age, 6 weeks) were purchased from the Experimental Animal Tech Co. of Weitonglihua (Beijing, China). All animal experiments were approved by the animal ethics committee of West China Hospital of Sichuan University (Ethic number 201403A). The TAC surgery is followed the protocol by our previous report (Zhou et al., 2020), which is widely used to establish animal models of pressure overload-induced cardiac fibrosis (Furihata et al., 2016). Briefy, the mice were anesthetized with isoflurane, then putted on the heating pad. The 27-gauge blunt needle was putted under the aortic arch and constricted with a silk suture (5–0), and then the needle was removed after tighten it up. Similar procedures without ligation were operated on Sham-group mice. At the end of procedure (28 days after operation), the mice were sacrificed and the hearts were collected for further experiments.

### Echocardiography Analyses

An echocardiography machine (35 MHz, Vevo3100, FUJIFILM) was applied to measure cardiac remolding of mice after TAC surgery (28 days). The mice were anesthetized by isoflurane and placed on operation pad in a supine position. Images were acquired of the left ventricle to measure the situation of cardiac remolding.

### Histological Analyses

After mice were sacrificed, the hearts of mice were excised freshly and fixed with 4% paraformaldehyde. The tissues were embedded in paraffin, and sectioned with 4–5 μm thickness. Heart sections were stained with hematoxylin-eosin, Masson’s trichrome or Sirius red staining following the protocol of manufacturers. Staining section images were captured using a Leica DMI3000B microscope. The level of cardiac fibrosis was analyzed by Image J software (the National Institutes of Health, NIH) by comparing the blue-stained area or red-stained area (collagen) with total area (Zhou et al., 2020).

### Cell Culture and Treatments

CFs were isolated from the hearts of neonatal C57BL mice (0–3 days after birth) according to the protocol reported previously (Sreejit et al., 2008). The CFs were grown in a culture plates with DMEM containing 10% fetal bovine serum (FBS) with 100 U/ml streptomycin and penicillin. CFs were cultivated to 80–90% confluence, and then treated with TGF-β1 (10 ng/ml, Sino biological Inc, China) for 24 h to induce CFs activation. For function study, the CFs were treated with 1 μM MRS2356 (Tocris, United States), an agonist of P2Y1R or 1 μM BPTU (Selleck, United States), an antagonist of P2Y1R. For cell transfection, CFs were transfected with siRNA (RiboBio, China) when cultivated to 50–60% confluence. SiRNA targeting P2Y1R were transfected into CFs for 24 h, which then treated with TGF-β1 for further 24 h. Transfection reagent (iMax, thermo, United States), siRNAs (100 nM) and MEM (Gibco, United States) were incubated at room temperature (RT), then added into culture plates. The sequences of each siRNA are shown in Supplement Table S1.

### Quantitative Real-Time PCR (qRT-PCR)

To detect the activation characteristics of CFs and fibrosis of hearts of mice, qRT-PCR assay was performed. Briefly, total RNA of was isolated from hearts of mice or cultured CFs using RNA isolation kit (Tianmobio, China) following the manufacturer’s instructions. Then, the RNA was used as templated to synthesize cDNA using a reverse transcription (RT) kit (Toyobo, Japan). The qRT-PCR was performed to detect gene expression using the SYBR Green Supermix kit (Bio-Rad, United States) on the CFX96 detection system (Bio-Rad, United States). The sequences of primers are shown in Supplement Table S2. The 2^−ΔΔCt^ threshold (Ct) method was applied to calculate relative fold changes. GAPDH served as the reference gene.

### Immunofluorescence Staining

Immunofluorescence staining of CFs was performed as previously reported (Xin et al., 2019). The cultured CFs were stained for α-SMA (Abcam, 1:200). Images were obtained with confocal microscopy (Zeiss, Germany). Six fields of view were randomly captured for each sample to calculated the α-SMA fluorescence intensity and analyzed using the ImageJ software.

### Western Blot

Total protein of heart tissues of mice and cultured CFs were obtained according to laboratory protocol using RIPA lysis buffer. Then BCA kit (Beyotime, China) was used for protein quantification. Equal quantity protein of tissues or cells lysates were separated through SDS-PAGE (10%) and subsequently transferred to PVDF membrane (0.45 μm) (Millipore, United States). Then, the membranes were blocked for 1 h with TBST (tris-buffered saline with 1% Tween20) containing 5% BSA. Subsequently, the membrane was incubated overnight at 4°C with the primary antibody: connective tissue growth factor (CTGF) (Abcam, 1:1,000), α-smooth muscle actin (α-SMA) (Abcam, 1:1,000), TGF-β (Abcam, 1:1,000), P2Y1R (HuaBio, 1:1,000), POSTN (Abcam, 1:1,000), collagen I (COL-1) (Abcam, 1:1,000), β-tubulin (Abcam, 1:1,000) and β-actin (Abcam, 1:1,000). After that, the membranes were washed with TBST for three times and incubated with HRP-conjugated secondary antibodies (anti-rabbit/mouse) (Zsgb Bio, 1:2,000) for 2 h at RT. The blots were incubated with enhanced chemiluminescence (ECL) kit (Bio-Rad, Japan) and captured using chemiluminescence machine. Finally, the protein intensities in stripes were measured by ImageJ software.

### EdU Proliferation Assay

The proliferation activity of CFs was detected using EdU (5-ethynyl-2-deoxyuridine) staining kit (Ribobio, China) following the instruction of manufacturers. Cells were incubated with 50 μmol/L EdU (2 h), nuclei were stained with DAPI (30 min). The number of EdU-positive cells and DAPI-stained cells were observed under a fluorescent microscope (Olympus, Japan). The percentage of EdU-positive cells was considered as cell proliferation rate.

### Data Statistics

Data was analyzed by the SPSS21.0 software (SPSS, Inc, Chicago, United States). Differences between two groups with unpaired data were analyzed using Student’s *t*-test; multiple comparisons of normally distributed data were analyzed using ANOVA followed by Bonferroni’s multiple comparison post-tests. All values are presented as mean ± SEM; *n* refers to the sample size. *p* < 0.05 was considered significant.

## Results

### Expression Pattern of P2Y Receptor Subtypes in TAC-Induced Cardiac Fibrosis Mice

**FIGURE 1:**
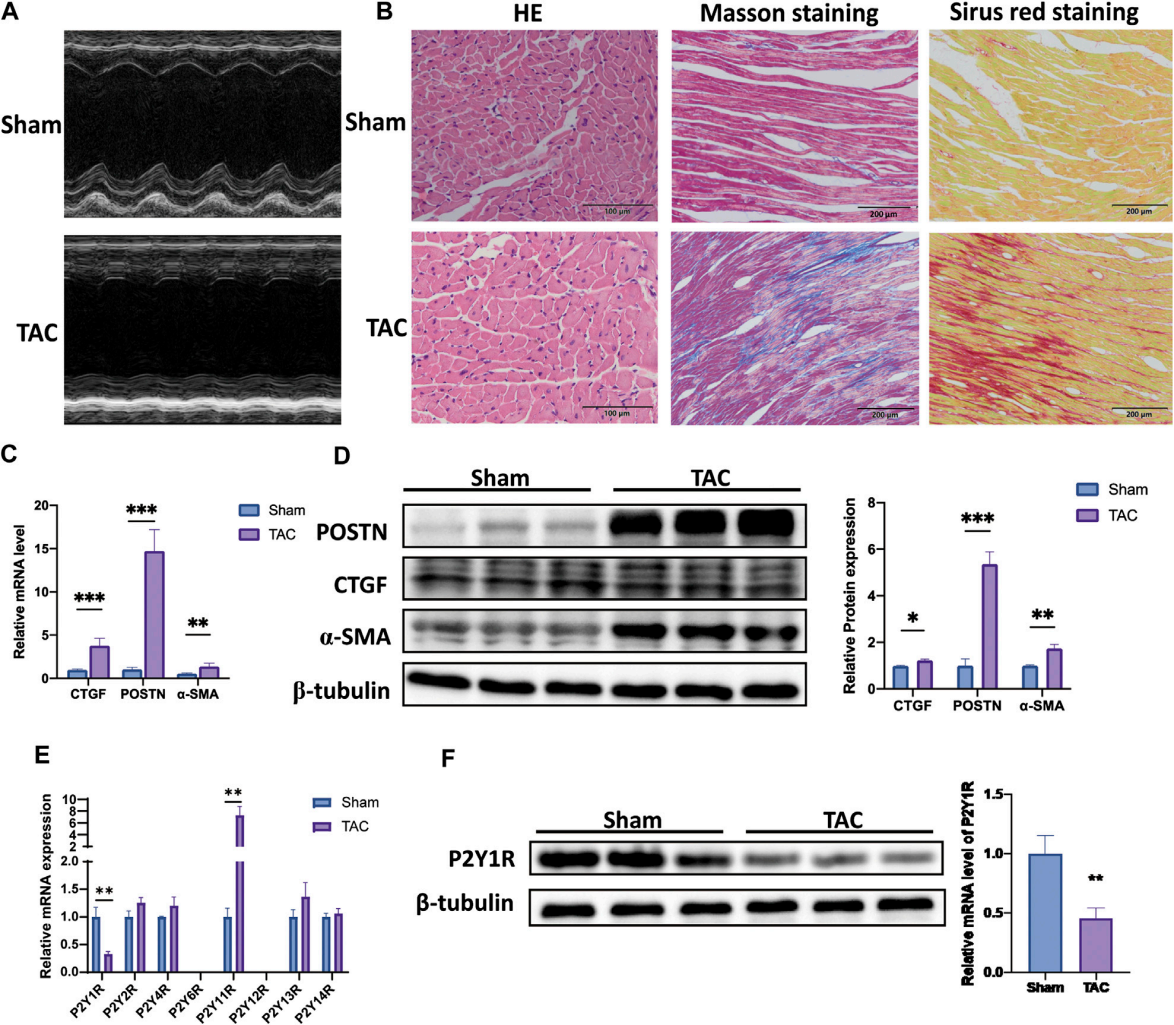
Expression pattern of P2Y receptor subtypes in TAC-induced cardiac fibrosis mice. **(A)** Representative image of an echocardiographic detection of mice left ventricle after TAC operation (4 weeks). **(B)** Representative image of HE-, Sirius Red-, and Masson’s trichrome-stained sections of mice after TAC operation. **(C)** Expression of mRNA of fibrosis markers (CTGF, POSTN, and α-SMA) in heart tissues of mice after TAC operation (*n* = 6). **(D)** Expression of protein of fibrosis markers (CTGF, POSTN and α-SMA) in heart tissues of mice after TAC operation (*n* = 6). **(E)** Gene expression of P2Y receptors subtypes in heart tissues of mice after TAC operation (*n* = 6). **(F)** Protein expression of P2Y1R in heart tissues of mice after TAC operation (*n* = 6). Results are presented as means ± standard deviation. ∗ indicates *p* < 0.05, ∗∗ indicates *p* < 0.01, and ∗∗∗ indicates *p* < 0.001.

Firstly, we established mice model with pressure overload-induced cardiac fibrosis by TAC operation. The cardiac remodeling with fibrosis was assessed by echocardiography detection, HE, Masson’s trichrome, and Sirius red staining (Figures 1A,B). The images showed that TAC mice had cardiac remolding, accompanying with a higher level of cardiac fibrosis and a larger area of cross-sectional. In addition, mRNA levels of CTGF, periosin (POSTN) and α-SMA in TAC mice were about 3.9, 14, and 2.1 times higher compared with sham mice, respectively (Figure 1C). The protein levels of CTGF, POSTN and α-SMA in TAC mice were also higher about 1.2, 5.3, and 1.7 times relative to sham mice (Figure 1D).

Subsequently, we explored the expression profile of P2Y receptor subtypes in left ventricle tissues by qRT-PCR assay. Only two P2Y receptor subtypes were significantly changed in TAC mice relative to sham mice, P2Y1R and P2Y11R. The mRNA expression of P2Y1R in TAC mice was decreased by nearly 33% compared to sham mice, while P2Y11R was higher about 10 times (Figure 1E).

### Expression Pattern of P2Y Receptor Family in TGF-β1- Induced Activation of CFs

**FIGURE 2:**
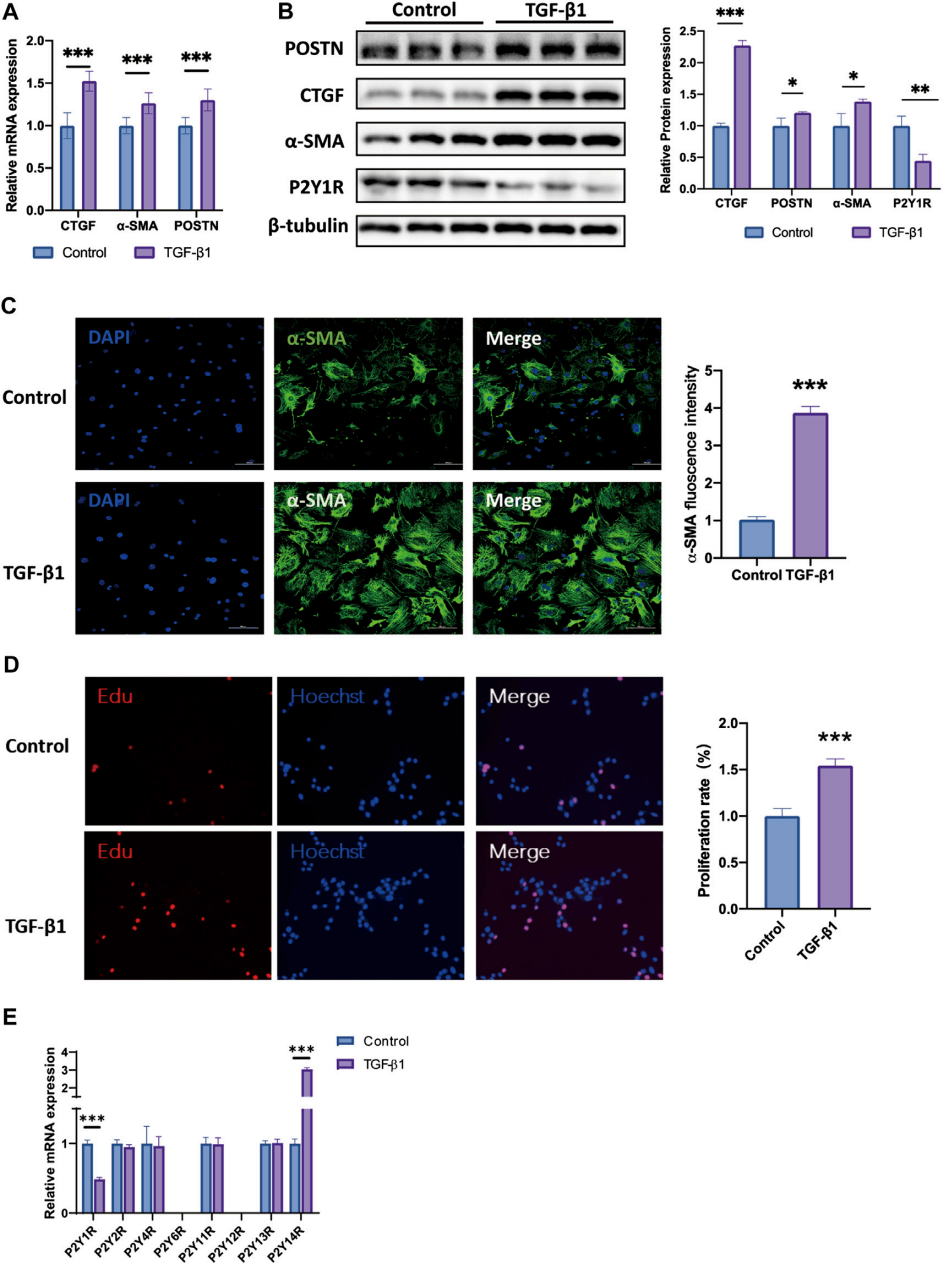
Expression pattern of P2Y receptor family in TGF-β1 induced activation of CFs. **(A)** Expression of CTGF, POSTN and α-SMA mRNAs in TGF-β1-induced activation CFs (*n* = 6). **(B)** Expression of CTGF, POSTN and α-SMA proteins in TGF-β1-induced activation CFs (*n* = 6). **(C)** Representative images of immunofluorescence staining of α-SMA (green) in CFs and nuclei DAPI (blue) (*n* = 6). **(D)** EdU staining images showing rate of cell proliferation. Proliferation cells stained with EdU (red) and nuclei stained with DAPI (blue) (*n* = 6). **(E)** Gene expression of P2Y receptors subtypes in TGF-β1-induced activation CFs (*n* = 6). Results are presented as means ± standard deviation. ∗ indicates *p* < 0.05, ∗∗ indicates *p* < 0.01, and ∗∗∗ indicates *p* < 0.001.

To confirm the P2Y receptor subtypes expression profiles, we established TGF-β1-induced activation of CFs *in vitro*. We detected the expression of profibrotic marker’s mRNAs in neonatal mice cardiac fibroblasts (CFs) after 24 h of TGF-β1 stimulation. The mRNA expressions of CTGF, α-SMA and POSTN, the known markers of cardiac fibrosis, in TGF-β1-induced CFs were higher about 1.5, 1.2, and 1.3 times than those in the control group, respectively (Figure 2A). In a similar trend, the protein levels of CTGF, POSTN, and α-SMA in TGF-β1-evoked CFs were also higher approximately 2.2, 1.2, and 1.4 times than in control group, respectively (Figure 2B). In addition, the immunofluorescence staining results showed that TGF-β1 stimulation enhanced the α-SMA intensities in CFs, and enlarged the α-SMA-expressed areas in CFs (Figure 2C). The number of EdU-positive CFs were 1.5-fold in TGF-β1-stimulated CFs than in control group (Figure 2D).

Then we explored the expression profile of P2Y receptor subtypes in CFs activation model by qRT-PCR assay. Only two P2Y receptor subtypes, P2Y1R and P2Y14R, were significantly changed in TGF-β1-stimulated CFs relative to control group. The mRNA expression of P2Y1R in TGF-β1-stimulated CFs was decreased by nearly 50% when compared to the control group, while P2Y14R was higher about 2.8 times (Figure 2E). The protein level of P2Y1R was also decreased by 55% in TGF-β1-induced CFs, compared to the control group (Figure 2B). Given the consistency of the *in vivo* and *in vitro* results, we then focused our study on P2Y1R and explored its function during the activation of CFs.

### P2Y1R Plays a Protective Role in the Process of CFs Activation

**FIGURE 3:**
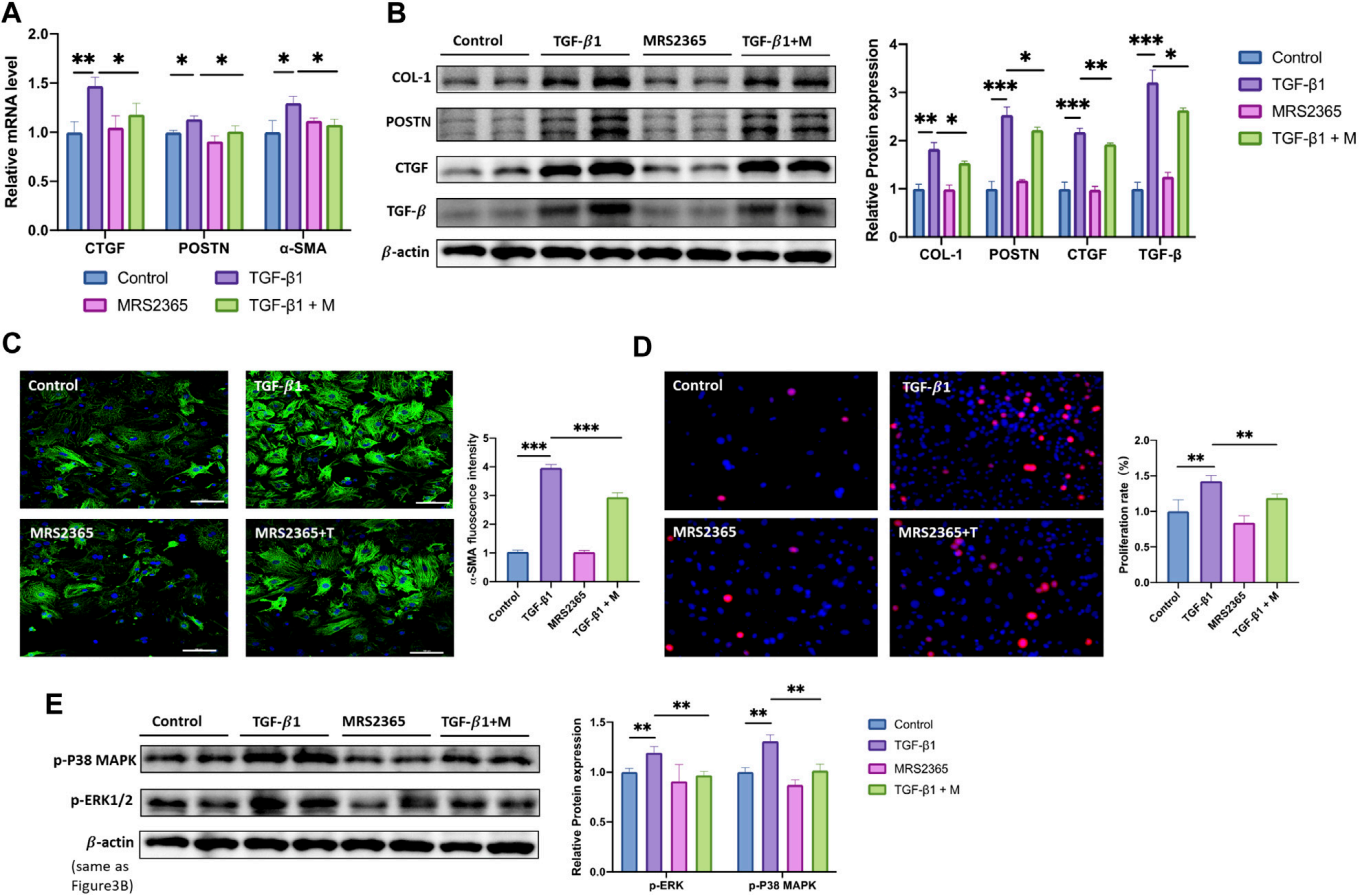
Activation of P2Y1R alleviates TGF- β1-stimulated CFs activation. **(A)** Expression of CTGF, POSTN and α-SMA mRNAs after CFs treated with TGF-β1 and MRS2365 (*n* = 6). **(B)** Protein levels of COL-1, POSTN, CTGF, and TGF-β after CFs treated with TGF-β1 and MRS2365 (*n* = 6). **(C)** Representative images of immunofluorescence staining of α-SMA (green) in CFs and nuclei DAPI (blue) (*n* = 6). **(D)** EdU staining images showing rate of cell proliferation. Proliferation cells stained with EdU (red) and nuclei stained with DAPI (blue) (*n* = 6). **(E)** Protein levels of p-P38 MAPK and p-ERK1/2 after CFs treated with TGF-β1 and MRS2365 (*n* = 6). Results are presented as means ± standard deviation. ∗ indicates *p* < 0.05, ∗∗ indicates *p* < 0.01, and ∗∗∗ indicates *p* < 0.001.

To investigate the function of P2Y1R in CFs activation stimulated by TGF-β1, MRS2365, an agonist of P2Y1R was employed in our study. Cultured CFs were treated with culture media with or without MRS2365 in the presence of TGF-β1 (24 h), and mRNA of profibrotic genes were measured by qRT-PCR and western blot assays. The results showed that, treatment with MRS2365 decreased mRNA expressions of CTGF (by 20%), POSTN (by 10%) and α-SMA (by 15%) compared with the TGF-β1 group (Figure 3A). The results of western blot also showed the similar trend with mRNA, that protein levels of COL-1, POSTN, CTGF, and TGF-β were reduced by 17, 12, 10, and 19% relative to the TGF-β1-treated CFs, respectively (Figure 3B and Supplement Figure 1). The result of immunofluorescence staining showed that MRS2365 administration significantly attenuated the increased florescence intensity of α-SMA induced by TGF-β1 (Figure 3C). In addition, the number of EdU-positive CFs in TGF-β1+ MRS2365 group was reduced by approximately 20%, compared to TGF-β1 group (Figure 3D). These results revealed that P2Y1R may play a protective role on the process of fibrosis and CFs activation stimulated by TGF-β1.

### Inhibition of P2Y1R Accelerates the Process of Fibrosis and CFs Activation

**FIGURE 4:**
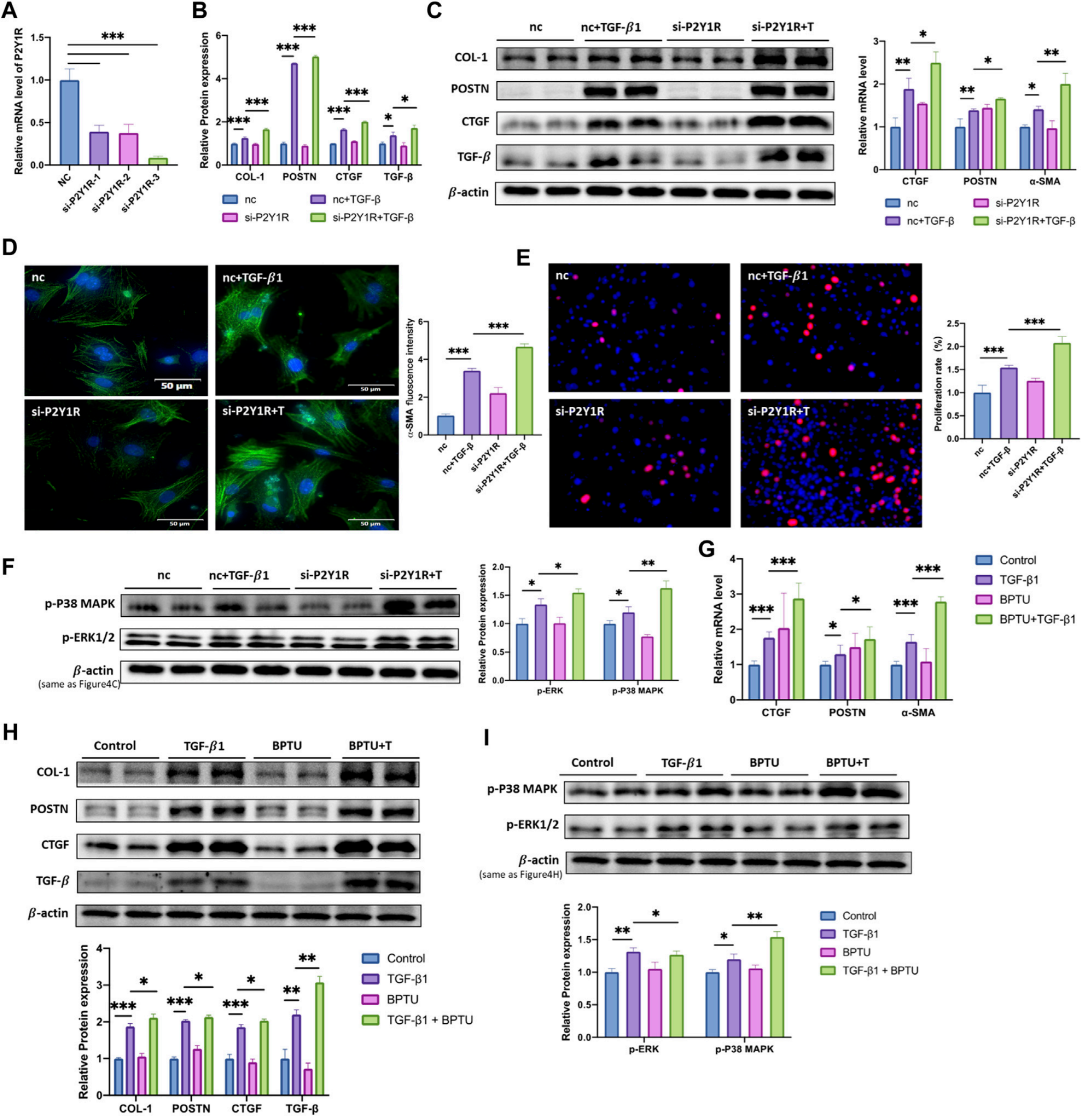
Inhibition of P2Y1R accelerates TGF- β1-induced CFs activation. **(A)** Expression of P2Y1R mRNA after si-RNA transfection (*n* = 3). **(B)** Expression of CTGF, POSTN and α-SMA mRNAs after CFs transfected with siRNA and treated with TGF-β1 (*n* = 6). **(C)** Protein levels of COL-1, POSTN, CTGF, TGF-β after CFs transfected with siRNA and treated with TGF-β1 (*n* = 6). **(D)** Representative images of immunofluorescence staining of α-SMA (green) in CFs and nuclei DAPI (blue) (*n* = 6). **(E)** EdU staining images showing rate of cell proliferation. Proliferation cells stained with EdU (red) and nuclei stained with DAPI (blue) (*n* = 6). **(F)** Protein levels of p-P38 MAPK and p-ERK1/2 after CFs transfected with siRNA and treated with TGF-β1 (*n* = 6). **(G)** Expression of CTGF, POSTN and α-SMA mRNAs after CFs treated with TGF-β1 and BPTU (*n* = 6). **(H)** Protein levels of COL-1, POSTN, CTGF, TGF-β after CFs treated with TGF-β1 and BPTU (*n* = 6). I Protein levels of p-P38 MAPK and p-ERK1/2 after CFs treated with TGF-β1 and BPTU (*n* = 6). Results are presented as means ± standard deviation. ∗ indicates *p* < 0.05, ∗∗ indicates *p* < 0.01, and ∗∗∗ indicates *p* < 0.001.

To further confirm the protective function of P2Y1R in process of fibrosis and CFs activation, specific siRNA of P2Y1R and BPTU, an inhibitor of P2Y1R, were applied in our study. CFs were transfected with si-P2Y1R, and the knockdown effect of si-P2Y1R was confirmed by qRT-PCR. The results revealed that, the most efficient si-P2Y1R sequence downregulated the P2Y1R mRNA expression by nearly 90% (Figure 4A). After transfection (24 h), TGF-β1 was applied. Compared with TGF-β1 group, down-regulated of P2Y1R turned the CFs into an further activation phenotype within CTGF, POSTN and α-SMA were increased 1.3-, 1.2- and 1.4- fold at mRNA level, while COL-1, POSTN, CTGF and TGF-β were increased 1.3-, 1.1-, 1.2- and 1.2- fold at protein level respectively (Figures 4B,C). Moreover, immunofluorescence images revealed upregulated expression of α-SMA by 1.4- fold compared with those in the TGF-β1 group (Figure 4D). Downregulating P2Y1R also promote the CFs proliferation revealed by the EdU results, showing an increase in the proliferation rate about 1.3-fold (Figure 4E).

The above results were consistent with the situation in the CFs treated with BPTU. Comparing with TGF-β1 group, BPTU administration increased the mRNA levels of profibrotic genes, CTGF, POSTN, and α-SMA were increased 1.7-, 1.4-, 1.6-fold respectively (Figures 4G). The western blot results revealed that BPTU-treated group had high protein levels of COL-1, POSTN, CTGF, and TGF-β about 1.1, 1.1, 1.2, and 1.4 times when compared with the TGF-β1 group (Figure 4H and Supplement Figure 2).

All above results demonstrated that P2Y1R might play a protective role in the process of fibrosis and CFs activation stimulated by TGF-β1.

### P2Y1R Modulates the CFs Activation Through p38 MAPK/ERK Signaling Pathway

As we have identified the role of P2Y1R in the process of CFs activation, we further determined the underneath mechanism. Given that p38 MAPK/ERK signaling pathway was involved in cardiac fibrosis (Deng et al., 2020b), we explored the activation of p38 and ERK signaling pathway in TGF-β1 treated CFs. Figure 3E showed that TGF-β1 stimulation induced the highly expressed of p-P38 MAPK and p-ERK1/2, which was attenuated by MRS2365 administration (*p* < 0.05). On the contrary, the treatment of BPTU or si-P2Y1R further increased phosphorylation of P38 MAPK and ERK1/2, which was consistent with the phenotype of activation of CFs (Figures 4F,I). Taken together, we drawn a crude conclusion that P2Y1R modulates CFs activation, at least partially, via p38 MAPK and ERK signaling pathway.

## Discussion

In this study, we investigated the expression pattern of P2Y subtypes in the process of cardiac fibrosis and CFs activation, and whether P2Y1R participated in these processes. In TAC model, the mRNA level of P2Y1R was decreased in TAC mice compared to sham mice, while P2Y11R mRNA level was higher in TAC mice. However, in CFs activation model, the P2Y11R mRNA level was no significant difference between control group and TGF-β1 group, while P2Y14R mRNA level was higher in TGF-β1 group than control group. Discordant results from *in vivo* and *in vitro* model, may be because heart tissues have other kinds of cells besides fibroblasts, such as myocytes, vascular endothelial cells and macrophages, and P2Y11R might expressed differently in these cells. Given the consistency of the *in vivo* and *in vitro* results, we focused our study on P2Y1R and explored its function during the activation of CFs.

Nucleotides, such as ATP and ADP, are secreted at a low level under physiological conditions (Vassort, 2001). However, a large number of ATP secreted into interstitial space when cells was under a hypoxia situation, high concentration of glucose or disturbed shear stress (Gombault et al., 2012; Novitskaya et al., 2016). Plenty of studies has demonstrated that ATP release contributes to inflammatory reactions and fibrosis in the injured cardiac tissues (Chen et al., 2012; Bracey et al., 2013; Zhao et al., 2016). Chen et al. reported that ATP activated P2X4/7 and P2Y2 receptors and up-regulated the proliferation of human CFs by promoting cell cycle progression (Chen et al., 2012). Similarly, our previous study showed that P2X7R was highly expressed in cardiac remolding and TGF-β1-evoked CFs activation. Inhibition of P2X7R with BBG alleviated the cardiac fibrosis induced by TAC operation *in vivo* (Zhou et al., 2020). Zheng et al. investigated the role of P2Y1R in norepinephrine (NE)-stimulated cardiac fibroblasts, revealed that P2Y1R modulates CFs proliferation via induction of c-fos and inhibition of DNA synthesis (Zheng et al., 1998; Szustak and Gendaszewska-Darmach, 2020). In addition, another study reported that A1, A2A, P2Y1R, P2Y11R and P2X7R agonists modulated the TGF-β1-evoked epithelial to mesenchymal transition (EMT) through the PKA and MAPK/ERK signaling pathway (Zuccarini et al., 2017). In our study, we established TAC-induced cardiac remolding (accompanying with hypertrophy and fibrosis) successfully. Our results demonstrated that P2Y1R was down-regulated in TAC operation mice and TGF-β1-induced activated CFs.

MRS2365, a highly potent and selective P2Y1R agonist is widely used to increase the content of calcium and dopamine in neurological diseases (Baker et al., 2018). This agonist displayed alternative function in P2Y1R-induced Ca^2+^ responses in spontaneously active urogenital tissues (Hashitani et al., 2020). Interestingly, the function of P2Y1R activation by MRS2365 displayed a double-edged sword effect. On one hand, Holger et al. revealed that activation of P2Y1R in medial prefrontal cortex impaired inhibitory control and behavioral flexibility (Koch et al., 2015). On the other hand, Lora et al. reported that treatment of mice with MRS2365 after trauma effectively reduced all post-injury symptoms of traumatic brain injury, including edema and neuronal swelling (Talley Watts et al., 2013). In our study, administration of the P2Y1R agonist MRS2365 alleviated the activation CFs stimulated by TGF-β1, whereas siRNA and inhibitors of P2Y1R treatment exhibited the opposite effect.

Numerous studies have reported the classical TGF-β1 signaling pathway (Smad mechanisms). In addition, more effects are needed to better clarify the role of mitogen-activated protein kinases (MAPKs), which plays pivotal roles in cell proliferation, differentiation and inflammation (Yamashita et al., 2008) (Cuadrado and Nebreda, 2010; Deng et al., 2020a). Previous studies have confirmed that MAPK signaling pathway are involved in fibrosis. Deng et al. revealed that peptide DR8 inhibits EMT by antagonizing the MAPK signaling to improve renal function, injury and fibrosis (Deng et al., 2020a). Zhou et al. reported that isorhamnetin alleviated liver fibrosis by inhibiting extracellular matrix formation via TGF-β1/p38 MAPK pathway (Liu et al., 2019). In our study, MAPK signaling pathways were activated in TGF-β1-treated CFs, showing increased levels of phosphorylation of ERK and p38 MAPK. On this basis, we explored whether MAPK signaling pathway activation were involved in P2Y1R activation process. As known as ERK, p38 kinases and JNK are the main subgroups of the MAPK family, we detected the levels of p38 and phosphorylation of ERK during P2Y1R activation process (Lamouille and Derynck, 2011). In addition, Franke et al. demonstrated that protective effect of P2Y1R against oxidative stress-induced cell death depends on whether level of ERK1/2 phosphorylation going back to its normal (Franke et al., 2009). Besides, blocking MAPK with a specific inhibitor had an inhibitory function in downregulating the level of α-SMA and ECM deposition (Andrikopoulos et al., 2019). Coincidentally, these reports were consistent with our study that MRS2365, an agonist of P2Y1R, inhibited the phosphorylation of ERK and p38 MAPK, alleviating the activation of CFs. In contrast, BPTU, an antagonist of P2Y1R, has a deleterious effect on the activation of CFs, and is accompanied with upregulation of ERK and p38 phosphorylation. To sum up, we preliminary demonstrated that MAPK/ERK signaling pathway might be activated during the process of cardiac fibrosis and CFs activation.

However, there are several limitations in this work. Whether agonists of P2Y1R, such as MRS2365, have *in vivo* potency against fibrosis needs to be confirmed in further study. Moreover, our present study only focused on the cardiac fibroblasts in the heart tissues, and the roles of P2Y1R in other cell types, such as cardiomyocytes, macrophages and endothelial cells are worth further exploring.

## Conclusion

In conclusion, our results demonstrate that P2Y1R is involved in TAC-induced cardiac fibrosis and TGF-β1-evoked CFs activation, and play a protective role. MAPK/ERK signaling pathway might be activated during this process. Our findings corroborate and expand previous studies of purinergic signaling in the process of cardiac fibrosis.

## Data Availability

The raw data supporting the conclusions of this article will be made available by the authors, without undue reservation.
