# Supplementary material for: P2Y1 Receptor Agonist Attenuates Cardiac Fibroblasts Activation Triggered by TGF-β1
Source: Front Pharmacol. 2021 Feb 17;12:627773. doi: 10.3389/fphar.2021.627773 (PMC7926204; doi:10.3389/fphar.2021.627773)
Supplement: Supplementary file 1 [file DataSheet1.docx]

Supplement

Supplement Table 1: Sequences of siRNA to P2Y1R

| Target | Sequences of siRNA |
| --- | --- |
| si-P2Y1R-1 | CTCATCTTCTACTACTTCA |
| si-P2Y1R-2 | TGTCTTACATCCCTTTCCA |
| si-P2Y1R-3 | GGTTCATCTTCCATGTAAA |
| si-NC | Provided by RiboBio^TM^ |

Supplement Table 2: Mice qRT-PCR primer sequences

| Target mRNA | Sequences (5’-3’) |
| --- | --- |
| CTGF | Forward: GGACACCTAAAATCGCCAAGC |
|  | Reverse: ACTTAGCCCTGTATGTCTTCACA |
| POSTN | Forward: TGGTATCAAGGTGCTATCTGCG |
|  | Reverse: AATGCCCAGCGTGCCATAA |
| α-SMA | Forward: GGACGTACAACTGGTATTGTGC |
|  | Reverse: TCGGCAGTAGTCACGAAGGA |
| P2Y1R | Forward: ACAAGACTGACTGGATCTTCGG |
|  | Reverse: TACACCACGCCACTGTACCT |
| GAPDH | Forward: AATGGATTTGGACGCATTGGT |
|  | Reverse: TTTGCACTGGTACGTGTTGAT |


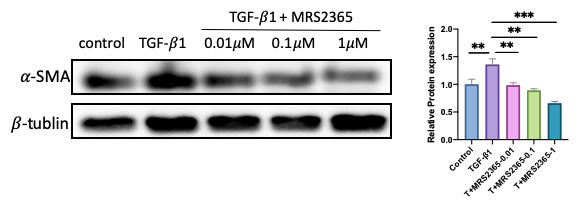


Supplement Figure 1: Protein expression of 𝛼-SMA of CFs after TGF-𝛽1 treatment with or without MRS2365 (n=6)

Results are presented as means ± standard deviation. ∗ indicates P < 0:05, ∗∗ indicates P < 0:01, and ∗∗∗ indicates P < 0:001.


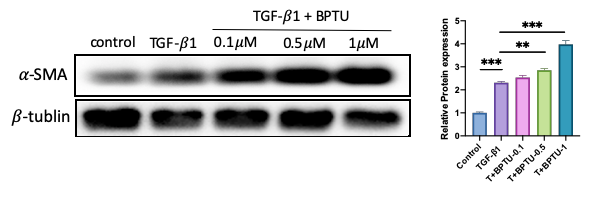


Supplement Figure 2: Protein expression of 𝛼-SMA of CFs after TGF-𝛽1 treatment with or without BPTU (n=6)

Results are presented as means ± standard deviation. ∗ indicates P < 0:05, ∗∗ indicates P < 0:01, and ∗∗∗ indicates P < 0:001.
